# Integrated analysis of metabolome and transcriptome reveals key candidate genes involved in flavonoid biosynthesis in *Pinellia ternata* under heat stress

**DOI:** 10.1007/s10265-023-01446-8

**Published:** 2023-03-07

**Authors:** Lianan Guo, Jun Tan, Xiaoshu Deng, Rangyu Mo, Yuan Pan, Yueqing Cao, Daxia Chen

**Affiliations:** 1 Chongqing Academy of Chinese Materia Medica, Chongqing, 400065 China; 2 Chongqing Sub-center of National Resource Center for Chinese Materia Medica, China Academy of Chinese Medical Science, Chongqing, 400065 China; 3 Chongqing Engineering Research Center for Fine Variety Breeding Techniques of Chinese Materia Medica, Chongqing, 400065 China; 4 School of Life Sciences, Chongqing University, Chongqing, 400044 China

**Keywords:** Flavonoid biosynthesis, Heat stress, Metabolome, *Pinellia ternata*, Transcriptome

## Abstract

*Pinellia ternata* (Thunb.) Breit. is an important traditional Chinese medicinal herb and very sensitive to high temperatures. To gain a better understanding of flavonoid biosynthesis under heat stress in *P. ternata*, we performed integrated analyses of metabolome and transcriptome data. *P. ternata* plants were subjected to a temperature of 38 °C, and samples were collected after 10 d of treatment. A total of 502 differential accumulated metabolites and 5040 different expressed transcripts were identified, with flavonoid biosynthesis predominantly enriched. Integrated metabolomics and transcriptome analysis showed that high temperature treatment upregulated the expression of *CYP73A* and downregulated the expression of other genes (such as *HCT, CCoAOMT*, *DFR1, DFR2*), which might inhibit the biosynthesis of the downstream metabolome, including such metabolites as chlorogenic acid, pelargonidin, cyanidin, and (-)-epigallocatechin in the flavonoid biosynthesis pathway. The transcription expression levels of these genes were validated by real-time PCR. Our results provide valuable insights into flavonoid composition and accumulation patterns and the candidate genes participating in the flavonoid biosynthesis pathways under heat stress in *P. ternata*.

**Supplementary Information:**

The online version contains supplementary material available at 10.1007/s10265-023-01446-8.

## Introduction

Global warming has led to frequent extreme high temperature events, which have become one of the main factors affecting plant yields. High temperature not only affects the phenotype of plants, but also destroys cell homeostasis, seriously affecting the growth and development of plants and even contributing to their death (Vinocur and Altman 2005). Research shows that heat stress can inhibit various physiological and biochemical reactions of plants, including changes in water status, cell membrane stability, photosynthesis, secondary metabolites, and plant hormone levels (Wahid et al. 2007). Therefore, it is important to unravel how plants respond to high temperature stress to improve the heat tolerance of plants.

*Pinellia ternata* (Thunb.) Breit. (known as ‘Banxia’ in Chinese) is an important perennial medicinal plant in the Araceae family with a long history of clinical application in China. The wild *P. ternata* populations are widely distributed in China, Korea, and Japan. *P. ternata* is very sensitive to high temperatures, which can cause the leaves of *P. ternata* to turn yellow and shrink, reducing its yield and quality (Xue et al. 2007). Its dried tuber has been widely used as a traditional medicine with antitussive, antiemetic, analgesic, antiobesity, antipyretic, expectorant, and sedative effects (Chen et al. 2003; Kim et al. 2006; Luo et al. 2000). Furthermore, the tubers of *P. ternata* can be used to treat anxiety, cancer, and inflammation and also to terminate early-stage pregnancies (Han et al. 2006; Hu et al. 2008).

The tubers of *P. ternata* are rich in chemical components, including alkaloids, flavonoids, organic acids, volatile oils, steroids, and sugars. Flavonoids, such as daidzein, apigenine, and baicalin, are important active ingredients in the tubers of *P. ternata* (Ji et al. 2014). Flavonoids are mainly derived from the flavonoid biosynthesis pathway, which originates from the phenylpropanoid pathway. Flavonoids have multiple biological functions, such as anti-oxidative, anti-diabetic, anti-inflammatory, and anti-hypertensive activities (Hossain et al. 2016; Ikarashi et al. 2018; Tejada et al. 2018). Previous studies found that key enzymes catalyze the biosynthesis of flavonoids, such as chalcone synthase (CHS), chalcone isomerase (CHI), flavonol synthase (FLS), flavanone 3-hydroxy-lase (F3H), flavonoid 3′-hydroxylase (F3′H), flavonoid 3′5′-hydroxylase (F3′5′H), dihydroflavonol 4-reductase (DFR), anthocyanidin synthase/leucocyanidin dioxygenase (ANS/LDOX), and flavonoid 3-*O*-glucosyltransferase (UFGT) (Pandey et al. 2016). Flavonoid metabolism is closely related to environment stress factors, such as drought, salt, pH, and light (Ma et al. 2014; Pi et al. 2018). In tea callus, flavonoids were remarkably down-regulated under light deprivation stress (Shi et al. 2020), and a similar phenomenon had been found in tea leaves under shade versus sunlight (Wang et al. 2012). In ripening fruit, drought conditions can increase anthocyanin accumulation (Castellarin et al. 2007). In soybean, 24 flavonoids were significantly up-regulated after salt treatment (Ma et al. 2014).

However, the current research on *P. ternata* mainly concentrates on the effects of physiological characteristics and chemical components, and very little research focuses on the molecular mechanism of accumulation of flavonoids under heat stress (Xue et al. 2004; Xue et al. 2007; Xue et al. 2021; Yahagi et al. 2021). Therefore, it is important to dissect the regulatory network of flavonoids in tubers of *P. ternata* under heat stress. In this study, we investigated flavonoid biosynthesis in tubers of *P. ternata* after 10 days of heat treatment using integrated analyses of transcriptome and metabolome data. The purpose of our study was to analyze the species of flavonoids and differentially expressed genes involved in flavonoid biosynthesis in the tubers of *P. ternata* under heat stress. Our results shed light on the molecular metabolism of flavonoid biosynthesis under heat stress, provide an empirical basis for further study of flavonoids, and will be helpful for breeding varieties with high flavonoid content under heat stress in *P. ternata.*

## Materials and methods

### Plant materials and high temperature treatment

Tubers of *P. ternata* were collected from Wanzhou district (30° 23′ 50″ to 31° 0′ 18″ N and 107° 52′ 22″ to 108° 53′ 52″ E), Chongqing, China and planted in an artificial climate chamber (MGC-HP-2 L, Yiheng, Shanghai, China) with a photoperiod of 14/10 h (day/night), temperature of 25/20 ℃ (day/night), and 65% relative humidity. For heat treatments, one-month-old seedings were transferred to 38/30 °C (day/night) conditions with a 14/10 h photoperiod and 65% relative humidity. *P. ternata* plants cultured at 25 °C with a 14/10 h photoperiod and 65% relative humidity were used as controls. After 10 d of heat treatment, tubers were harvested and cleaned. All tuber samples were immediately frozen in liquid nitrogen and stored at − 80 °C.

### Metabolic profiling

Metabolite extraction and profiling analysis were performed by Tianjin Novogene Bio-Tech Co., Ltd. (www.novogene.com) following the company’s standard procedures and as previously fully described by Wang et al. (2017). A total of 100 mg of the freeze-dried tubers was individually grounded with liquid nitrogen (MM 400; Retsch, Haan, Germany), and the homogenate was resuspended with prechilled 80% methanol and 0.1% formic acid by well vortex. The samples were incubated on ice for 5 min and then were centrifuged at 15,000×*g*, 4 °C for 20 min. Some supernatant was diluted to a final concentration containing 53% methanol with LC-MS grade water. After centrifugation at 15,000×*g* and 4 °C for 20 min, the supernatant was filtered through a 0.22 μm microporous membrane (SCAA-104, ANPEL, Shanghai, China) for LC-MS/MS analysis.

LC-MS/MS analysis was performed using a UHPLC system (Vanquish, Thermo Fisher, Dreieich, Germany) coupled with a mass spectrometer (Orbitrap Q Exactive™ HF-X, Thermo Fisher), which was equipped with a Hypesil Gold column (100 × 2.1 mm, 1.9 μm). The injection volume was 4 µL at a flow rate of 0.2 mL min^− 1^. The mobile phase was a solution of ultrapure water (with 0.1% formic acid) and methanol. The solvent gradient was set as follows: 98:2 0.1% formic acid:methanol, 1.5 min; 0:100 0.1% formic acid:methanol, 12.0 min; 0:100 0.1% formic acid:methanol, 14.0 min; 98:2 0.1% formic acid:methanol, 14.1 min; 98:2 0.1% formic acid:methanol, 17.0 min.

The raw data were processed using Compound Discoverer 3.1 (CD3.1, Thermo Fisher) to perform peak alignment, peak picking, and quantitation for each metabolite. Analyst 1.6.1 software (AB SCIEX, Ontario, Canada) was used to analyze the metabolite data. These metabolites were annotated using the Kyoto Encyclopedia of Genes and Genomes (KEGG) database (https://www.genome.jp/kegg/pathway.html), Human Metabolome Database (HMDB) (https://hmdb.ca/metabolites), and LIPID Maps database (http://www.lipidmaps.org/). Principal component analysis (PCA) and partial least squares discriminant analysis (PLS-DA) were carried out to identify differentially expressed flavonoids using metaX (Wen et al. 2017). Univariate *t*-tests were used to calculate the statistical significance (*P*-value). The metabolites with variable importance for projection (VIP) > 1 and fold change ≥ 2 or ≤ 0.5 were considered to be differential metabolites.

### Transcriptome sequencing, assembly, and functional annotation

The total RNA extraction kit (TIANGEN, Beijing, China) was used to extract total RNA from the tubers of *P. ternata*. Total RNA concentration and purity were measured by the Nano Photometer spectrophotometer (IMPLEN, Westlake Village, CA, USA), and the RNA quality was analyzed using the Qubit RNA Assay Kit in Qubit 2.0 Fluorometer (Life Technologies, CA, USA). RNA integrity was assessed using the RNA Nano 6000 Assay Kit of the Bioanalyzer 2100 system (Agilent Technologies, South San Francisco, CA, USA). A total amount of 1 µg of RNA per sample was used as input material for the RNA sample preparations. Using this input material, mRNA was randomly fragmented into smaller pieces, and six RNA sequencing libraries were constructed using NEBNext Ultra RNA Library Prep Kits for Illumina (NEB, Ipswich, MA, USA) according to the product instructions. The clustering of the index coded samples was performed on a cBot Cluster Generation System using the TruSeq PE Cluster Kit v3-cBot-HS (Illumina, San Diego, CA, USA) according to the manufacturer’s instructions. After cluster generation, sequencing was performed on an Illumina HiSeq platform at Tianjin Novogene Bio-Tech Co., Ltd. (www.novogene.com).

To acquire high-quality reads, strict quality control of the data was conducted. Clean reads were obtained by removing reads containing adapter sequences, reads containing N base calls, and low-quality reads from the raw data. Trinity software (v2.4.0) was used to splice and assemble clean reads to obtain transcripts and unigenes (Grabherr et al. 2011). The functions of all unigenes were annotated based on sequence similarities to sequences in six public databases, including the Nr, Nt, Pfam, COG, Swiss-Prot, KEGG, and GO databases. After predicting the amino acid sequence of unigenes, the annotation of unigenes was obtained by comparing them with the Pfam database using HMMER software (v3.3).

Gene expression levels were estimated using RSEM software (v1.2.15). Differential expression analysis between different sample groups was performed using the DESeq2 package (1.20.0). The false discovery rate (FDR) was obtained by multiple hypothesis testing for a hypothesis testing probability (*P*-value) using the Benjamini and Hochberg approach. Genes with log_2_ (fold change) > 1.5 and FDR < 0.05 were assigned as differentially expressed. The KEGG pathway of each DEG was obtained from the KEGG website (https://www.genome.jp/kegg). The hypergeometric test was used to find the KEGG pathways that were significantly enriched for DEGs compared to the whole genome background.

### qRT-PCR analysis of candidate genes

RNA samples were prepared by the total RNA extraction kit (TIANGEN, Beijing, China), and cDNAs were then synthesized using the M5 Super Plus qPCR RT Kit with gDNA Remover kit (Mei5bio, Beijing, China) for qRT-PCR. Samples were amplified with ChamQ™ Universal SYBR qPCR Master Mix SYBR (Vazyme, Nanjing, China) and detected by the CFX connect Real-time PCR system (Bio-Rad, Hercules, CA, USA). Three biological replicates were performed for each treatment, and three replicates were performed for each sample. The specific primers were designed using Primer Premier 5.0, and the primer sequences are listed in Table S2. Primer specificity was verified by melting curve analysis. The *P. ternata GAPDH* gene was used as the reference transcript. The qRT-PCR analyses were conducted as described previously (Kenneth and Thomas 2001). The levels of gene expression relative to those in control conditions were quantified as relative quantification (RQ) values, which were calculated using the 2^−ΔΔCt^ method.

## Results

### Metabolic differences between control and heat treatment samples

**Fig. 1:**
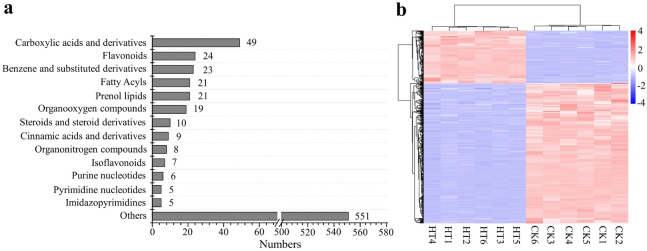
Number of metabolites by type of compound and heatmap of metabolite profiles of two groups of samples. **a **Number of different types of metabolites in all samples. **b** Clustering heatmap of the metabolites detected in all samples. Each example is visualized in a single column, and each metabolite is represented by a single row. Red indicates high abundance, whereas low relative abundance metabolites are shown in blue

To further study the effects of heat stress on secondary metabolites, the sample extracts were analyzed based on the widely targeted metabolomics approach by using UPLC-ESI-MS/MS and public and self-built databases together (including a MetWare database). A total of 758 secondary metabolites were detected in control (CK) and heat treatment (HT) samples, including 49 carboxylic acids and derivatives, 24 flavonoids, 23 benzene and substituted derivatives, 21 fat acyls, 21 prenol lipids, 19 organooxygen compounds, 10 steroids and steroid derivatives, 9 cinnamic acids and derivatives, 8 organonitrogen compounds, 7 isoflavonoids, 6 purine nucleotides, 5 pyrimidine nucleotides, 5 imidazopyrimidines, and 551 other metabolites (Fig. 1a). A hierarchical heatmap clustering analysis of the samples was performed using the metabolite concentration data (Fig. 1b). We observed that all the biological replicates were grouped together (top side of the figure), indicating high reliability of the generated metabolome data. In particular, we observed clear separation between CK and HT samples, suggesting that the metabolite characteristics were obviously distinct in these two samples.

### Identification of the differentially accumulated metabolites between CK and HT samples

**Fig. 2:**
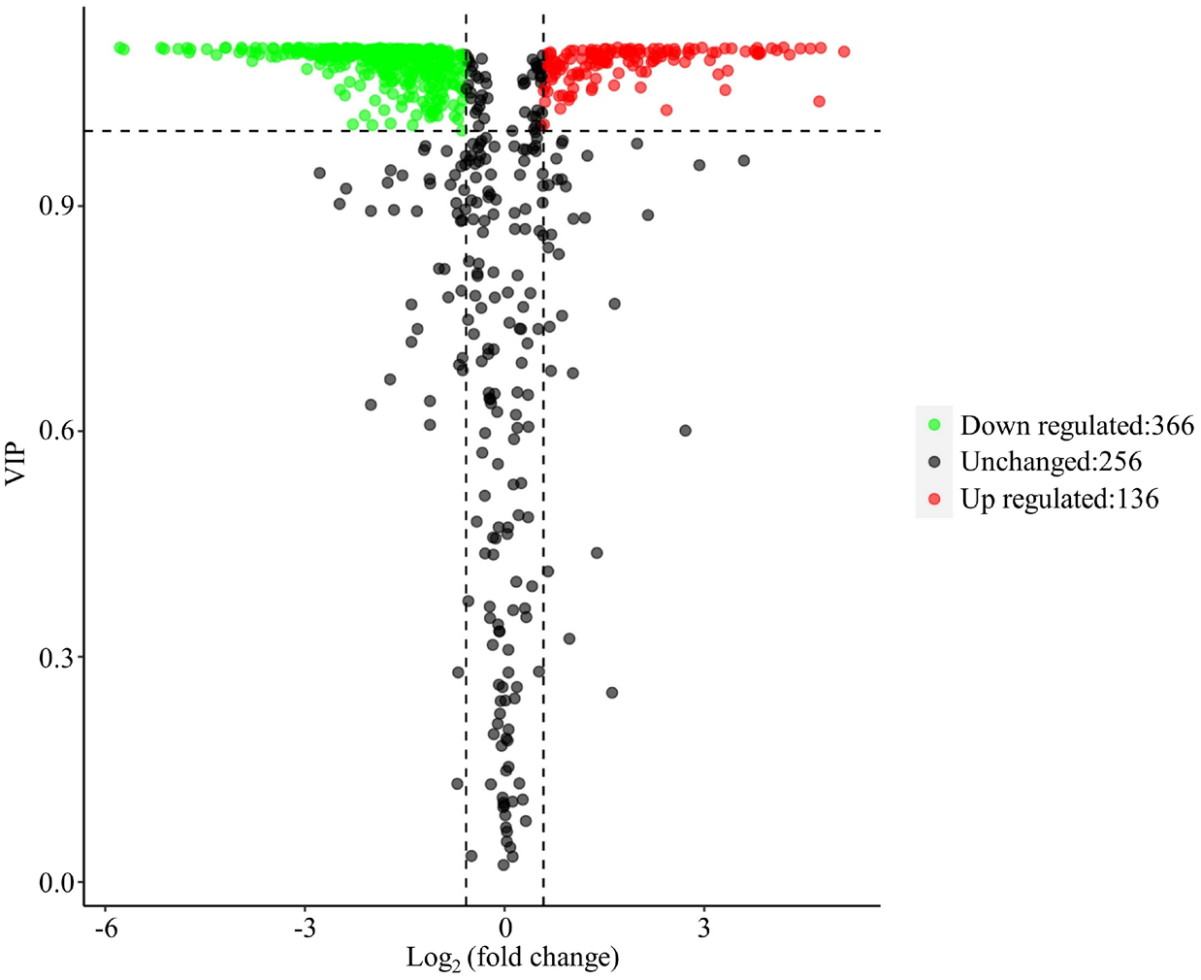
Variable importance for projection (VIP) plot of differentially accumulated metabolites (DAMs) in the comparison of control (CK) vs. heat treatment (HT) samples.Each dot represents a metabolite. Significantly upregulated metabolites in HT are indicated by red dots, and significantly downregulated metabolites in HT are indicated by green dots. DAMs were defined as those with a fold change ≥ 2 or ≤ 0.5 and VIP > 1.

**Fig. 3:**
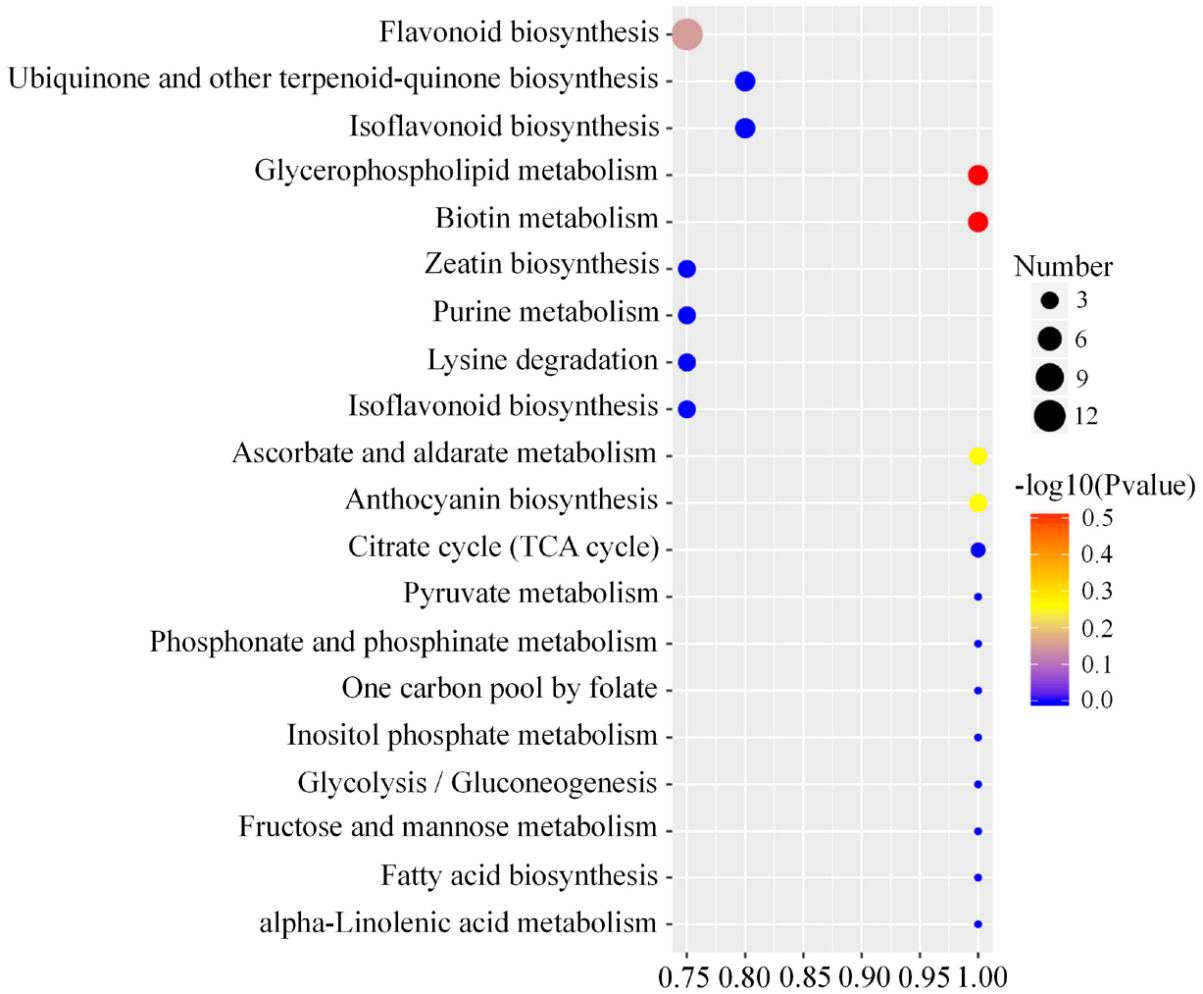
Kyoto Encyclopedia of Genes and Genome (KEGG) pathway enrichment of all differentially accumulated metabolites in the comparison of control (CK) vs. heat treatment (HT) samples. The 20 most significant categories with the lowest corrected *P*-values are shown

The differentially accumulated metabolites (DAMs) between CK and HT samples were screened based on the criteria of variable importance in projection (VIP) ≥ 1 and fold change ≥ 2 or ≤ 0.5. As expected, 502 metabolites were differentially accumulated between CK and HT samples, with 136 increased and 366 decreased under high temperature stress compared with the control (Fig. 2). To further understand which metabolic pathways were activated under high temperature stress, all differentially accumulated metabolites were mapped to KEGG pathways. Based on KEGG analysis, a total of 29 metabolic pathways were identified. KEGG enrichment analysis showed that 11 metabolites were mapped to flavonoid biosynthesis (ko00941), and 4 metabolites were mapped to isoflavonoid biosynthesis (ko00943) (Fig. 3; Table 1, S4). Furthermore, all metabolites annotated to flavonoid and isoflavonoid biosynthesis were decreased in HT samples compared with CK samples.

**Table 1:** List of differentially expressed genes (DEGs) in heat treatment (HT) vs. control (CK) samples in the flavonoid biosynthesis pathway under high temperature stress

Index	Metabolite name	KEGG ID	CK Content	HT Content	log_2_FC
Com_5980	Naringenin	C00509	5,992,764	930,074	− 2.69
Com_2634	Pelargonidin	C05904	27,618,730	5,758,093	− 2.26
Com_1993	Vitexin	C01460	41,744,267	8,901,970	− 2.23
Com_4640	Apigenin	C01477	10,354,634	2,662,688	− 1.96
Com_2428	Chlorogenic acid	C00852	29,616,849	7,768,919	− 1.93
Com_10546	Naringenin chalcone	C06561	1,630,210	444,958	− 1.87
Com_5211	Naringin	C09789	8,071,383	2,628,666	− 1.62
Com_7999	Prunin	C09099	3,379,338	1,248,458	− 1.44
Com_1544	Hesperetin	C01709	58,561,491	23,955,822	− 1.29
Com_3251	Cyanidin	C05905	16,654,035	8,212,537	− 1.02
Com_4216	(-)-Epigallocatechin	C12136	12,007,248	7,696,250	− 0.64

### Transcriptome analysis of CK and HT samples

In this study, RNA extracted from the tuber tissues of *P. ternata* was chosen to explore the differences in gene expression between CK and HT samples by transcriptome sequencing. The overview of the RNA-seq reads for six libraries is listed in Table 2. A total of 133,016,944 clean reads were obtained from six libraries. After data assembly, a total of 121,812 unigenes with N50 and N90 lengths of 1015 bp and 383 bp were detected, all of which were longer than 300 bp, as shown in Table S1. In total, 54,726 unigenes were between 300 and 500 bp in length, accounting for 44.93% of all unigenes (Table S1). The results suggested that the quality of the output data was good and that bioinformatics analysis could be conducted. All unigenes were annotated for gene function annotation in seven databases, including NR, NT, KOG/COG, PFAM, Swiss-Prot, KEGG, and GO. Of all unigenes, 4331 and 60,881 were annotated in all databases and in at least one database, accounting for 3.55% and 49.97% of unigenes, respectively (Table 3).

**Table 2:** Sequencing quality of transcriptome of *Pinellia ternata* under high temperature stress

Sample	Raw reads	Clean reads	Clean Bases (G)	N (%)	Q20 (%)	Q30(%)	GC (%)
CK1	23,848,426	23,639,906	7.09	0.02	97.99	94.46	55.01
CK2	22,670,726	22,424,337	6.73	0.02	97.93	94.35	54.84
CK3	22,248,348	21,941,700	6.58	0.02	98.05	94.49	55.45
HT1	20,616,506	20,346,142	6.1	0.03	97.89	94.17	53.1
HT2	23,652,376	23,402,364	7.02	0.03	97.82	93.98	53.05
HT3	21,433,344	21,262,495	6.38	0.02	97.96	94.26	52.53
Total	134,469,726	133,016,944	39.90	0.02	97.94	94.29	54.00

**Table 3:** Successful gene annotation statistics

Database	Number of genes	Percentage (%)
Annotated in NR	46,991	38.57
Annotated in NT	24,930	20.46
Annotated in KEGG	16,676	13.68
Annotated in Swiss-Prot	33,007	27.09
Annotated in PFAM	36,640	30.07
Annotated in GO	36,640	30.07
Annotated in KOG	8908	7.31
Annotated in all Databases	4331	3.55
Annotated in at least one Database	60,881	49.97
Total Unigenes	121,812	--

**Fig. 4:**
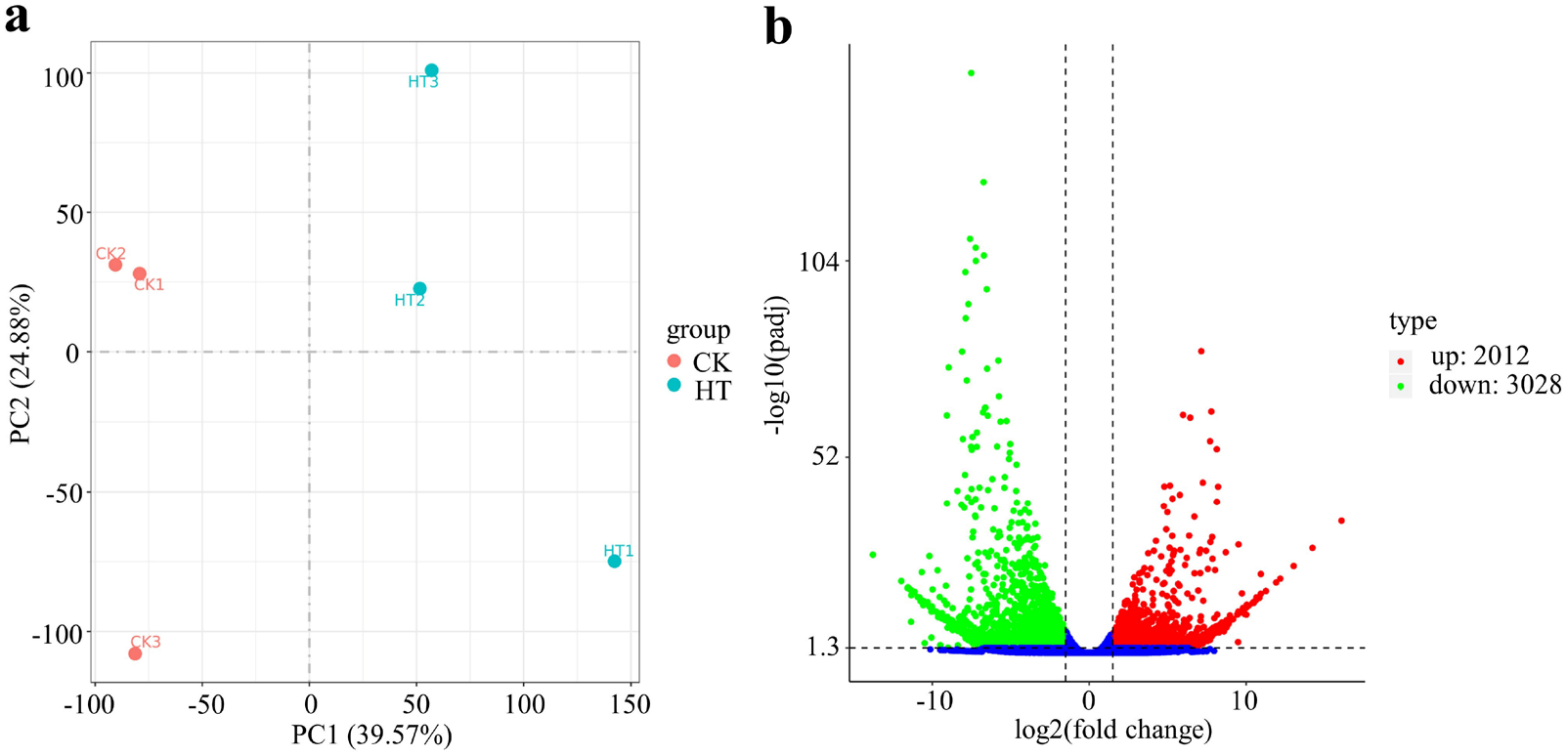
Difference in the expression patterns between two temperatures for ***Pinellia ternata ***tubers. **a** 2D principal component analysis (PCA) score plot of CK vs. HT. **b** Volcano plot of differentially regulated genes in the comparison of control (CK) vs. heat treatment (HT) samples. The red dots were expressed more in HT, and green dots were expressed more in CK.

PCA based on the fragments per kilobase of exon per million fragments mapped (FPKM) values was employed to identify differences in expression profiles among samples (Fig. 4a). The HT and CK samples were clearly distinguished by the first principal component (PC1, explaining 39.57% of all variation), indicating the expression patterns of unigenes differed between HT and CK samples. To remove the effects of length differences and sequencing depth, the expression level of all unigenes was calculated using the FPKM method (Trapnell et al. 2010). DEGs were defined as genes that were significantly enriched or depleted in one sample relative to another according to DESeq^2^ (with threshold criteria of log_2_ (fold change) > 1.5 and FDR < 0.05). In total, 5040 DEGs were identified between CK and HT samples, with 2012 up-regulated and 3028 down-regulated DEGs in HT samples, respectively (Fig. 4b).

### KEGG enrichment of DEGs between CK and HT samples

**Fig. 5:**
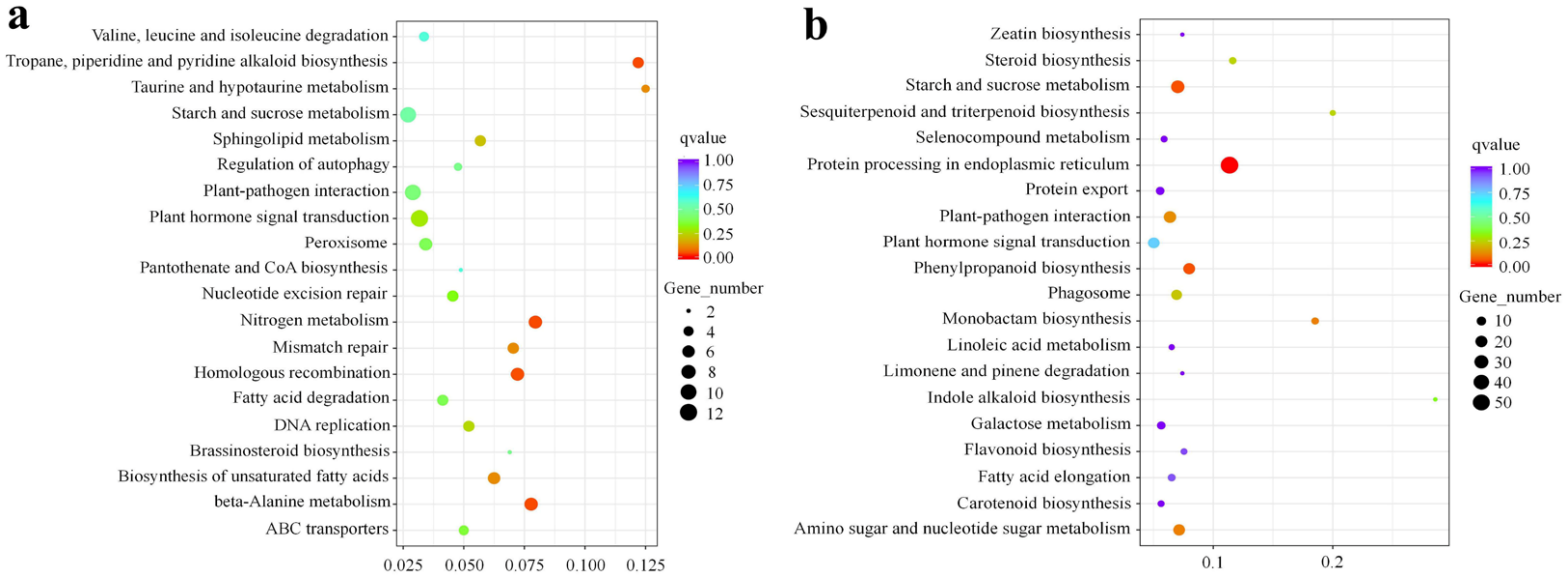
Top 20 Kyoto Encyclopedia of Genes and Genomes (KEGG) pathways enriched for differentially expressed genes (DEGs) between control (CK) and heat treatment (HT) samples.** a** DEGs expressed more in HT; **b** DEGs expressed more in CK. The dot color represents the *q*-value, and the dot size represents the number of DEGs.

For functional annotation, KEGG pathway enrichment analysis was carried out by KOBAS 2.0 software. A total of 493 DEGs were annotated to 104 KEGG pathways. The results for the top 25 KEGG pathways enriched in each group are represented as a histogram in Fig. S1, including flavonoid biosynthesis (ko00941). Among the five DEGs annotated to flavonoid biosynthesis (ko00941), four DEGs were down-regulated and one DEG was up-regulated in HT samples (Fig. 5). Notably, flavonoid biosynthesis (ko00941) was significantly enriched in the KEGG analysis of down-regulated DEGs (Fig. 5b). This result preliminarily indicated obvious differences in the above substances between CK and HT samples.

### Integrated analysis of metabolites and transcripts in the flavonoid biosynthesis pathway

**Fig. 6:**
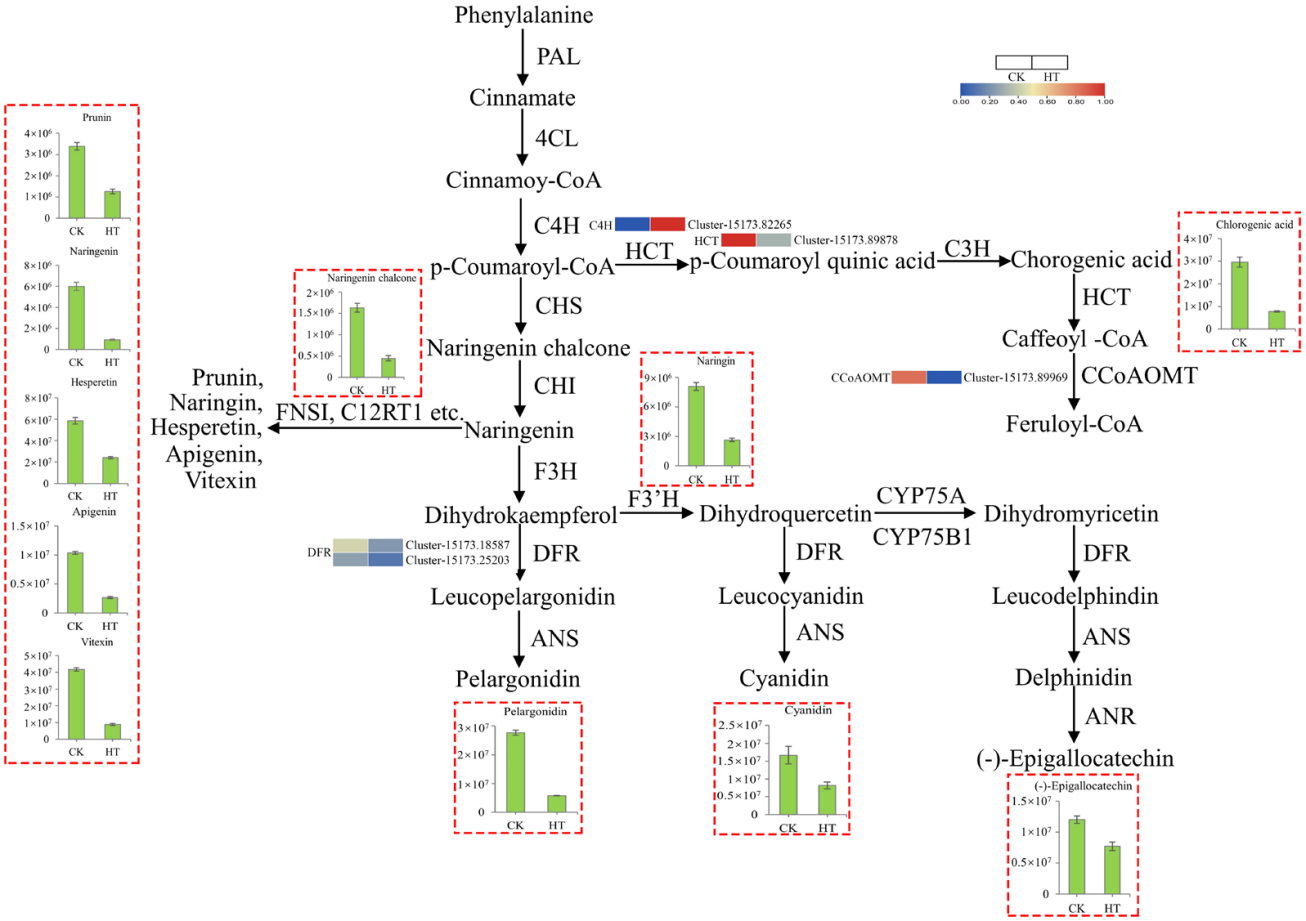
Transcriptional regulation under high temperature stress in the flavonoid biosynthesis pathway. The histogram displays the levels of 11 differentially accumulated metabolites (The histogram of flavonoids is expressed as relative content). Error bars represent ± standard error (*n* = 6). Heatmap showing the expression patterns of the candidate genes involved in the regulation of flavonoid compounds in tubers of *Pinellia ternata* under heat stress. Color scale indicates FPKM value. *C4H* cinnamic acid 4-hydroxylase, *HCT* shikimate O-hydroxycinnamoyltransferase, *CCoAOMT* caffeoyl-CoA O-methyltransferase, *DFR* dihydroflavonol 4-reductases

Combined analysis showed metabolites and transcripts participating in 23 KEGG reference pathways altogether, including flavonoid biosynthesis, which revealed distinct metabolism patterns between CK and HT samples (Fig. S2). The DAMs, together with the DEGs, are indicated on the flavonoid biosynthesis pathways in Fig. 6. In the flavonoid biosynthesis pathways, the expression of *CYP73A* (cinnamic acid 4-hydroxylase) was up-regulated, while naringenin chalcone and naringenin and its derivatives accumulated less under high temperature stress. The expression of *HCT* was down-regulated; at the same time, chlorogenic acid accumulated less under high temperature stress. The expression of *DFR1* and *DFR2* decreased, and the contents of pelargonidin, cyanidin, and (-)-epigallocatechin decreased remarkably. In addition, the expression of *CCoAOMT* also decreased, but no differentially accumulated metabolites were detected. The FPKM values of the DEGs mentioned above are illustrated in Table S3, and the abundance of the DAMs and DEGs mentioned above is presented in Table 1 and 4, respectively.

**Table 4:** List of differentially accumulated metabolites (DAMs) in heat treatment (HT) vs. control (CK) samples in the flavonoid biosynthesis pathway under high temperature stress

Accession Number	Function Prediction	KEGG ID	CK Count	HT Count	log_2_FC
Cluster-15173.82265	CYP73A	K00487	6.13	64.22	3.38
Cluster-15173.89878	HCT	K13065	333.61	19.16	-4.12
Cluster-15173.89969	CCoAOMT	K00588	25.28	0	-7.31
Cluster-15173.18587	DFR1	K13082	282.36	26.19	-3.43
Cluster-15173.25203	DFR2	K13082	196.14	14.88	-3.72

### qRT-PCR analysis of genes involved in flavonoid biosynthesis

**Fig. 7:**
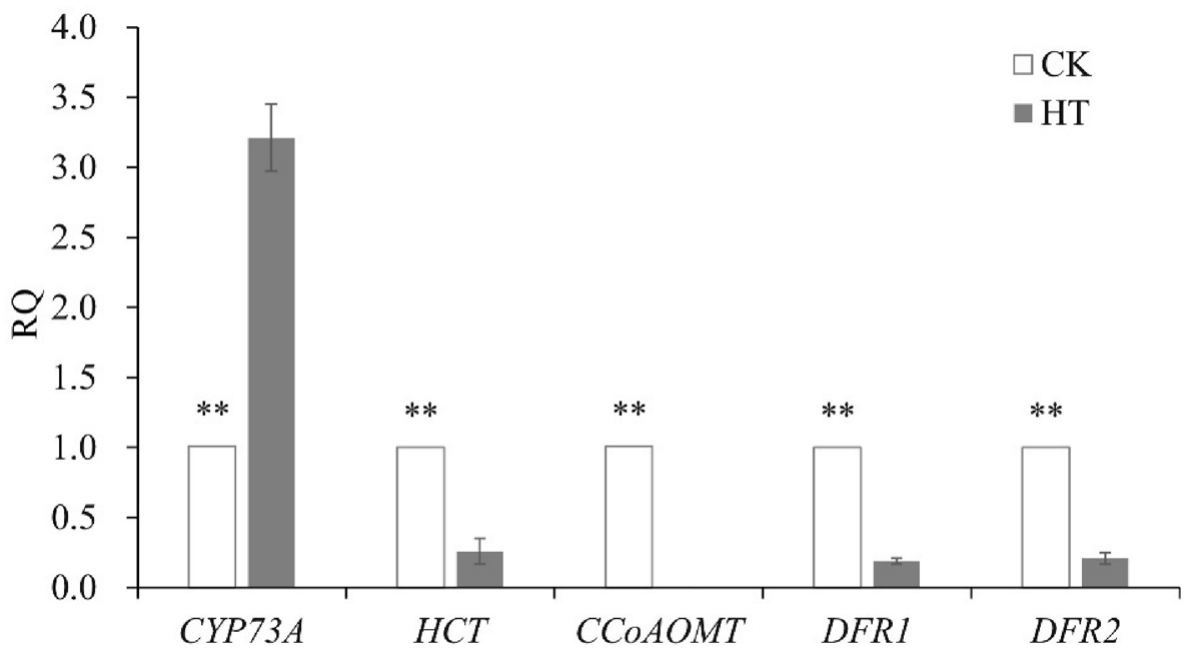
Relative expression of flavonoid biosynthesis-related genes in control (CK) and heat treatment samples (HT). Total RNA was extracted from *Pinellia ternata* tubers on day 10 after control and high temperature treatments. The *P. ternata GAPDH* gene was used as an internal reference. The gene expression levels of HT samples relative to those in CK samples are represented by relative quantification (RQ) values, which were calculated using the 2^−ΔΔCt^ method. Values are means of three biological replicates. Error bars indicate ± SE. Data are presented as means ± SE (*n* = 3). Statistical significance was determined by *t*-tests. ** indicates a significant difference (*P* < 0.01)

The expression of DEGs involved in the flavonoid biosynthesis pathway was verified by qRT-PCR (Fig. 7). Significantly reduced transcript levels were detected for the following four DEGs participating in flavonoid biosynthesis in HT samples, including *HCT* (predicted to encode a shikimate *O*-hydroxycinnamoyltransferase that catalyzes phenylpropanoids in the production of coniferyl and sinapyl alcohols), *CCoAOMT* (predicted to encode a caffeoyl-CoA *o*-methyltransferase that contributes to lignin deposition), and *DFR1* and *DFR2* (predicted to encode dihydroflavonol 4-reductases that are key enzymes involved in the late steps of the anthocyanin biosynthetic pathways). In HT samples, the expression level of *CYP73A*, which was predicted to encode a cinnamic acid 4-hydroxylase that catalyzes the hydroxylation of trans-cinnamic acid into *trans-p*-coumaric acid, was three times greater than that in CK samples.

## Discussion

As global warming has continued to intensify in recent years, short-term and long-term extreme high temperature events are occurring frequently, causing serious impacts on plant yield. High temperature stress can change plant morphology, physiology, and gene expression and inhibit the normal growth and development of plants. Improving the heat tolerance of plants has become a key focus of research. To date, the effects of high temperature on transcripts and metabolites were rarely reported in the flavonoid biosynthesis pathway in *P. ternata*. Transcriptome analysis showed that a total of 5040 DEGs were identified under high temperature, with 2012 up-regulated and 3028 down-regulated DEGs. Among these identified DEGs, five DEGs were annotated to the flavonoid biosynthesis pathway. Most of DEGs involved in the flavonoid biosynthesis pathway were down-regulated under high temperature stress; at the same time, the content of 11 metabolites mapped to flavonoid biosynthesis accumulated less under high temperature treatment. These results indicated that flavonoid biosynthesis was inhibited by high temperature in *P. ternata*.

Two transcripts annotated to flavonoid biosynthesis (ko00941) and predicted as *HCT* and *CCoAOMT* were down-regulated most under high temperature stress. The expression level of *HCT* decreased 94.26%, and *CCoAOMT* was not expressed under high temperature stress. Consistent with the transcriptome data, qRT-PCR demonstrated four transcripts were less expressed, and one transcript was more expressed under high temperature stress in the flavonoid biosynthesis pathway; among these transcripts, the expression of *HCT* decreased by nearly four times. These qRT-PCR results confirmed the changes in the expression levels of five genes under high temperature stress.

CYP73A (cinnamic acid 4-hydroxylase) is a cytochrome P450 monooxygenase associated externally with the endoplasmic reticulum in plant cells (Zhang et al. 2020). CYP73A uses NADPH-cytochrome P450 reductase as a donor of electrons and hydroxylates cinnamic acid to form 4-coumaric acid (Mizutani and Ohta 2010; Rustgi et al. 2019). In tobacco (*Nicotiana tabacum*), alfalfa (*Medicago sativa*), and *Arabidopsis thaliana*, downregulation of *C4H* (*CYP73A*) led to a decrease in lignin content (Acker et al. 2013; Blount et al. 2000; Sewalt et al. 1997). In this study, the expression of *CYP73A* was induced by high temperature treatment and suppressed the accumulation of downstream metabolites, including naringenin chalcone, naringenin, prunin, naringin, hesperetin, apigenin, and vitexin, which were decreased in HT samples, suggesting that high temperature stress leads to high expression of *CYP73A*, which may inhibit the biosynthesis of flavonoids. In addition, the expression of *CCoAOMT* decreased dramatically, but no DAMs were detected in the flavonoid biosynthesis pathway.

Chlorogenic acid is generated as an important intermediate product in the phenylpropanoid biosynthesis pathway. As a widespread dietary component, chlorogenic acid widely exists in many natural products, such as tea, coffee, and fruits (Mikami and Yamazawa 2015). Previous studies have reported that chlorogenic acid has multiple medicinal effects, including anti-oxidative and anti-inflammatory effects and reducing the incidence of several chronic and degenerative diseases (Liang and Kitts 2015; Santana-Gálvez et al. 2017; Upadhyay and Rao 2013). The biosynthesis of chlorogenic acid is regulated differently in plants. In *Artemisia annua*, decreased expression of *C4H* led to reduced accumulation of cinnamic acid and p-coumaric acid (Kumar et al. 2016). In *Arabidopsis thaliana*, a mutation in C4H results in the accumulation of cinnamoylmalate (Schilmiller et al. 2009). In the HT sample, the content of chlorogenic acid was decreased; at the same time, the expression of *HCT* was significantly down-regulated. Thus, these results suggest that increased expression of HCT may contribute to the accumulation of chlorogenic acid in *P. ternata*.

In the flavonoid biosynthesis pathway, dihydroflavonol 4-reductase (DFR) is a key rate-limited enzyme that controls the carbon flux direction of the anthocyanin pathway and participates in the biosynthesis of anthocyanins, proanthocyanidins, and other flavonoids that are essential for plant survival and human health (Miyagawa et al. 2015; Xie et al. 2004). In many plants, DFR proteins catalyze three substrates, including dihydrokaempferol, dihydromyricetin, and dihydroquercetin, into eucopelargonidin, leucocyanidin, and leucodelphinidin, respectively (Ni et al. 2020). Previous studies have shown that a single *DFR* gene is typically found in multiple plant species (Bongue-Bartelsman et al. 1994; Chen et al. 1998; Holton and Cornish 1995; Shirley et al. 1992). Although multiple *DFR* genes have been identified, only one *DFR* gene has catalytic activity in several plants, such as morning glory (*Ipomoea purpurea*) and Gerbera hybrids (Helariutta et al. 1993; Johnson et al. 2001). In the present study, two *DFR* genes, *DFR1* and *DFR2*, were detected by transcriptome analysis in *P. ternata*. The expression of *DFR1* and *DFR2* was down-regulated in HT samples and appeared to suppress the accumulation of pelargonidin, cyanidin, and (-)-epigallocatechin, the product of leucopelargonidin, leucocyanidin, and leucodelphindin in the flavonoid biosynthesis pathway, respectively. It remains unclear whether only one or both of the DFR proteins are catalytically active in *P. ternata*. In Arabidopsis *tt3-1* mutants, overexpressing *OjDFR1* successfully restored the deficiency of anthocyanin and proanthocyanidins (Sun et al. 2021). In pink-leaved ornamental kale, overexpression and virus-induced gene silencing verified *BoDFR1* conferred anthocyanin accumulation (Feng et al. 2021). These results suggested that the two *DFR* genes may play a key role in the biosynthesis of anthocyanins under high temperature stress. Regulatory genes involved in anthocyanin accumulation were identified in this study, providing a new strategy for regulation of anthocyanin content in *P. ternata*.

In summary, total flavonoid biosynthesis was influenced by heat stress in tubers of *P. ternata*. We identified changes in the flavonoid biosynthesis metabolic pathway in *P. ternata* tuber based on metabolome and transcriptome data. A total of 5040 DEGs and 11 flavonoid metabolites were identified to have differential accumulation under heat stress. Genes related to flavonoid biosynthesis were identified by integrated analysis of metabolome and transcriptome data from the tubers of *P. ternata*. These results provide valuable information on flavonoid compositions and accumulation patterns as well as the candidate genes participating in the flavonoid biosynthesis pathways under heat stress in *P. ternata*.

## Electronic supplementary material

Below is the link to the electronic supplementary material.

Supplementary file1 457 kb

## Data Availability

The whole set of raw data can be found in the national center for biotechnology information (NCBI) SRA database (accession number PRJNA917809).
