# Correction to: Integrated analysis of metabolome and transcriptome reveals key candidate genes involved in flavonoid biosynthesis in *Pinellia ternata* under heat stress

**DOI:** 10.1007/s10265-023-01458-4

**Published:** 2023-04-15

**Authors:** Lianan Guo, Jun Tan, Xiaoshu Deng, Rangyu Mo, Yuan Pan, Yueqing Cao, Daxia Chen

**Affiliations:** 1 Chongqing Academy of Chinese Materia Medica, Chongqing, 400065 China; 2 Chongqing Sub-center of National Resource Center for Chinese Materia Medica, China Academy of Chinese Medical Science, Chongqing, 400065 China; 3 Chongqing Engineering Research Center for Fine Variety Breeding Techniques of Chinese Materia Medica, Chongqing, 400065 China; 4 School of Life Sciences, Chongqing University, Chongqing, 400044 China

**Journal of Plant Research**

10.1007/s10265-023-01446-8

In the original publication of the article, titles of Tables 1, 4 were published incorrectly.

In the title of Table 1, “differentially expressed genes (DEGs)” should read as “differentially accumulated metabolites (DAMs)”. Likewise, in the title of Table 4, “differentially accumulated metabolites (DAMs)” should read as “differentially expressed genes (DEGs)”.

