# Supplementary material for: Integrated analysis of metabolome and transcriptome reveals key candidate genes involved in flavonoid biosynthesis in Pinellia ternata under heat stress
Source: J Plant Res. 2023 Mar 7;136(3):359–69. doi: 10.1007/s10265-023-01446-8 (PMC10126072; doi:10.1007/s10265-023-01446-8)
Supplement: Supplementary file 1 — Supplementary file1 457 kb [file 10265_2023_1446_MOESM1_ESM.pdf]

Electronic supplementary materials

Title:

Integrated Analysis of Metabolome and Transcriptome Reveals Key Candidate Genes Involved in Flavonoid Biosynthesis in *Pinellia ternata* under Heat Stress

Authors:

Lianan Guo[1,2,3,#], Jun Tan[1,2,3,#], Xiaoshu Deng[1,#], Rangyu Mo[1,2,3], Yuan Pan[1,2,3], Yueqing Cao[4], Daxia Chen[1,2,3,*]

[1] Chongqing Academy of Chinese Materia Medica, Chongqing 400065, China;

[2] Chongqing Sub-center of National Resource Center for Chinese Materia Medica, China Academy of Chinese Medical Science, Chongqing 400065, China;

[3] Chongqing Engineering Research Center for Fine Variety Breeding Techniques of Chinese Materia Medica, Chongqing 400065, China

[4] School of Life Sciences, Chongqing University, Chongqing 400044, China

Journal:

Journal of Plant Research

Corresponding author:

Daxia Chen

(Chongqing Academy of Chinese Materia Medica, 34 Nanshan Road, Nanan District, Chongqing, China)

Tel: +86-023-89029016

E-mail: 17837@163.com

Content:

Tables S1–S4

Figs. S1–S2

Table S1 Assembly statistics of transcriptome data from *Pinellia ternata* samples under high temperature stress.

length range/bp	Transcript		Unigene	
	Number	Percentage/%	Number	Percentage/%
300~500	123624	37.58	54726	44.93
500~1000	120215	36.55	39671	32.57
1000~2000	62615	19.04	19685	16.16
> 2000	22457	6.83	7730	6.34
Total	328911	-	121812	-
Total length	283651496	-	98409778	-
Mean length	862	-	808	-
Length of N50	1079	-	1015	-
Length of N90	414	-	383	-

Table S2 Primers used for RT-qPCR analysis.

Gene	NCBI Accession No.	Organism	Forward primer (5'-3')	Reverse primer (5'-3')
GAPDH	OMO60415.1	*Corchorus capsularis*	TGCTGGGAATGATGTTGAATG	TTGGCATTGTTGAGGGTTTG
CYP73A	XP_006850963.1	*Amborella trichopoda*	AGAAAGACCCGTCGGAGTTC	CAATGTGGAGGCAAGAACCG
HCT	RWR97947.1	*Cinnamomum micranthum*	CTTCTCCTTGAGCTTGCCGA	GAGTTCCGGCGCCATGTTAG
CCoAOMT	AEV43321.1	*Acacia auriculiformis*	GACGCCCTGTACCAATACATCT	GGGGAGTTGGCAGATCTTGA
DFR1	RWR80738.1	*Cinnamomum micranthum*	GCTGGATTCGTGGCTTCCTAT	TGGATCAGATCGGACGGTG
DFR2	XP_010926084.1	*Elaeis guineensis*	AGCAAGTTTGAACGGGGAGT	ACCAGTTGGACAAAGTGACAGA

Table S3 The fpkm of the five differentially expressed genes (DEGs) in *Pinellia ternata* tuber under heat stress.

Genes	Fpkm Value	
	CK	HT
CYP73A	0.45	6.70
HCT	27.28	2.07
CCoAOMT	22.68	0
DFR1	11.84	1.49
DFR2	7.09	0.72

Table S4 Relative content of differentially accumulated metabolites (DAMs) in *Pinellia ternata* tuber under heat stress.

Index	Compounds	CK1	CK2	CK3	CK4	CK5	CK6	HT1	HT2	HT3	HT4	HT5	HT6
Com_5980	Naringenin	5.64E+06	6.78E+06	5.84E+06	6.10E+06	5.71E+06	5.89E+06	1.00E+06	8.63E+05	8.44E+05	9.07E+05	1.03E+06	9.31E+05
Com_2634	Pelargonidin	2.76E+07	2.86E+07	2.77E+07	2.65E+07	2.88E+07	2.66E+07	5.55E+06	5.90E+06	5.97E+06	5.72E+06	5.73E+06	5.66E+06
Com_1993	Vitexin	4.04E+07	4.23E+07	4.30E+07	4.09E+07	4.30E+07	4.09E+07	9.97E+06	9.14E+06	8.89E+06	8.51E+06	8.18E+06	8.72E+06
Com_4640	Apigenin	1.03E+07	1.05E+07	9.98E+06	1.07E+07	1.05E+07	1.02E+07	2.40E+06	2.70E+06	2.85E+06	2.56E+06	2.53E+06	2.93E+06
Com_2428	Chlorogenic acid	3.11E+07	2.76E+07	3.07E+07	2.83E+07	2.69E+07	3.31E+07	8.23E+06	7.75E+06	7.88E+06	7.48E+06	7.39E+06	7.89E+06
Com_10546	Naringenin chalcone	1.63E+06	1.79E+06	1.58E+06	1.73E+06	1.48E+06	1.58E+06	4.48E+05	5.73E+05	3.84E+05	4.09E+05	4.16E+05	4.40E+05
Com_5211	Naringin	8.42E+06	7.97E+06	7.69E+06	8.10E+06	7.56E+06	8.70E+06	2.68E+06	2.88E+06	2.74E+06	2.61E+06	2.55E+06	2.31E+06
Com_7999	Prunin	3.33E+06	3.63E+06	3.24E+06	3.61E+06	3.30E+06	3.17E+06	1.42E+06	1.32E+06	1.22E+06	1.27E+06	1.17E+06	1.09E+06
Com_1544	Hesperetin	6.03E+07	5.99E+07	5.18E+07	6.12E+07	5.81E+07	6.01E+07	2.62E+07	2.35E+07	2.41E+07	2.29E+07	2.39E+07	2.31E+07
Com_3251	Cyanidin	1.32E+07	2.02E+07	1.58E+07	1.92E+07	1.69E+07	1.47E+07	7.02E+06	7.57E+06	9.85E+06	8.18E+06	7.64E+06	9.02E+06
Com_4216	(-)-Epigallocatechin	1.09E+07	1.21E+07	1.26E+07	1.18E+07	1.20E+07	1.27E+07	7.80E+06	7.52E+06	8.69E+06	6.44E+06	7.64E+06	8.08E+06

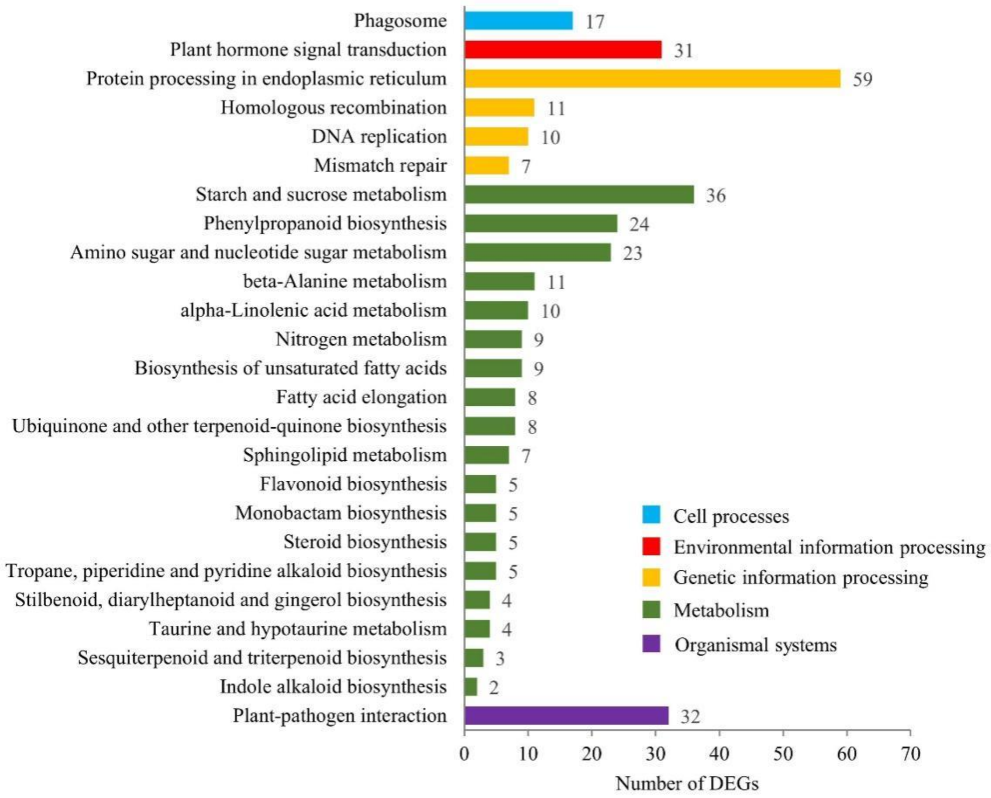

Fig. S1 Top 25 of metabolic pathways enriched for differentially expressed genes (DEGs) under high temperature stress treatment.

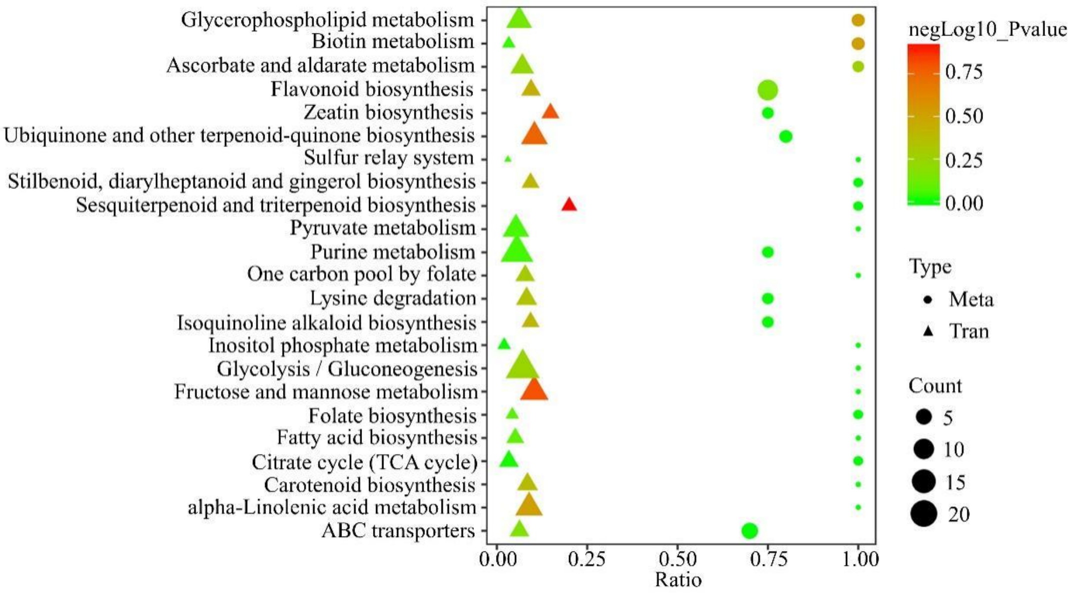

Fig. S2 KEGG enrichment analysis of differentially accumulated metabolites (DAMs) and differentially expressed genes (DEGs) under high temperature stress. Each count represents the number of DAMs or DEGs in the enriched pathway. The size of each dot or triangle represents the number of DAMs or DEGs, respectively. The abscissa represents the ratio of the number of differential DAMs or DEGs enriched in the pathway to the number of metabolites or genes annotated in the pathway.
